# Prevalence of and factors associated with hypertension according to JNC 7 and ACC/AHA 2017 guidelines in Bangladesh

**DOI:** 10.1038/s41598-021-94947-2

**Published:** 2021-07-29

**Authors:** Md. Ashfikur Rahman, Henry Ratul Halder, Uday Narayan Yadav, Sabuj Kanti Mistry

**Affiliations:** 1 Development Studies Discipline, Social Science School, Khulna University, Khulna, 9208 Bangladesh; 2 Statistics Discipline, Science, Engineering and Technology School, Khulna University, Khulna, 9208 Bangladesh; 3 Rady Faculty of Health Sciences, Department of Community Health Sciences, University of Manitoba, Winnipeg, Manitoba Canada; 4 Forum for Health Research and Development, Dharan, Nepal; 5 Centre for Primary Health Care and Equity, University of New South Wales, Sydney, Australia; 6 BRAC James P Grant School of Public Health, BRAC University, 68 Shahid Tajuddin Ahmed Sharani, Mohakhali, Dhaka, 1212 Bangladesh

**Keywords:** Diseases, Health care, Medical research, Risk factors

## Abstract

Most studies either followed Joint National Committee 7 (JNC 7) or World Health Organization-International Society of Hypertension (WHO-ISH) guidelines to ascertain the prevalence of hypertension among Bangladeshi adults. The American College of Cardiology/American Heart Association (ACC/AHA) revised the definition of hypertension in 2017, which has significant public health importance. In Bangladesh, the new guideline has resulted changes in prevalence and risk factors for hypertension compared to the JNC7 guideline. This study used data from the most recent round (2017–2018) of the Bangladesh Demographic and Health Survey (BDHS). According to the 2017 ACC/AHA guideline, the participants were categorized as hypertensive if they had blood pressure (BP) ≥ 130/80 mmHg, but it was ≥ 140/90 mmHg in JNC 7 guideline. A total of 11,959 participants were involved in the analysis. The median (IQR) age of the respondents was 34.0 (18.0–95.0) years. The prevalence of hypertension was 24.0% according to the JNC 7 guideline, which was 50.5% according to the 2017 ACC/AHA guideline. Participants who were overweight and obese, aged, member of affluent households, Rangpur and Rajshahi division inhabitants had significantly higher odds of being hypertensive according to both guidelines. The new guideline suggests that half of the adult population in Bangladesh is hypertensive when measured according to the new guideline, urging the policymakers and public health practitioners to take immediate action to address the already established modifiable risk factors.

## Key points

### Question

What changes occurred in prevalence of and associated factors with hypertension among Bangladeshi adults (aged 18–95 years) due to 2017 ACC/AHA and JNC 7 guidelines?

## Introduction

Globally, cardiovascular diseases (CVDs) are considered the leading causes of deaths or disability-adjusted life years, where hypertension plays a pivotal role in CVDs^1–3^. In 2016, around 17.9 million people died from CVDs, representing 31% of total global deaths, of which 9.4 million deaths were attributed to hypertension^4,5^. Worldwide, approximately 1.13 billion people have hypertension, and two-thirds of them live in low- and middle-income countries (LMICs), including Bangladesh^4^.

Due to the recent epidemiologic and demographic transitions, Bangladesh has documented significant lifestyle and behavioral changes with an increased prevalence of hypertension. According to the 2011 Bangladesh Demographic and Health Survey (BDHS) and the 2010 Non-Communicable Disease Risk Factor Survey, the prevalence of hypertension among the adult population was 25.7% and 17.9%, respectively^6,7^. Henceforth, hypertension remains the foremost disease burden among the major non-communicable diseases (NCDs) in Bangladesh, like other South Asian countries such as India, Nepal, Bhutan, and Sri Lanka^8^.

The blood pressure (BP) threshold to classify prehypertension and hypertension varies according to different guidelines. Previously, the Seventh Report of the Joint National Committee (JNC 7) on Prevention, Detection, Evaluation and Treatment of High Blood Pressure described hypertension as systolic blood pressure (SBP) of ≥ 140 mmHg and/or diastolic blood pressure (DBP) ≥ 90mmHg^9^. The 2017 American College of Cardiology/American Heart Association (ACC/AHA) Guideline for Prevention, Detection, Evaluation, and Management of High Blood Pressure in Adults reduced the BP threshold for hypertension. According to the 2017 ACC/AHA guideline, hypertensive individuals have a SBP of ≥ 130 mmHg and/or a DBP of ≥ 80 mmHg^10^. Therefore, previously considered prehypertensive participants are reclassified as hypertensive due to changes in the cut-off values^6,11–13^. For example, the prevalence of hypertension among the Nepalese adult population almost doubled following the 2017 ACC/AHA guidelines^12^.

Furthermore, Muntner et al. reported a 14.7% increase in hypertension among American adults over 19 years of age according to new guideline^14^. Another study found 45.1% and 26.8% increase in the prevalence of hypertension among adults (aged between 45 and 75) in the US and China^15^. Several studies recognized the importance of this revised classification for public health resource planning and prevention strategies^14–16^. Despite the increased burden of CVDs in developing countries, there remains inadequate information around the new guideline of hypertension^17–19^.

Previous studies in Bangladesh reported a significant increase in hypertension when the 2017 ACC/AHA guideline is being implemented to identify hypertensive individuals. Kibria et al. found that the prevalence of hypertension among Bangladeshi adults aged ≥ 35 years increased from 25.7 to 48.0%^6^. Islam et al. reported the findings from 1843 Bangladeshi adults aged over 18 years and found a similar increase (22.8%) in hypertension^20^. Besides, Kibria et al. concluded that the risk factors and their level vary between the JNC 7 and ACC/AHA guidelines^11^. Thus, changes in prevalence and risk factors have direct and indirect implications and hold significant merit in revising public health policies and plans to address the issue^21^. However, all these studies reported the findings from the 2011 BDHS survey data. A recent research article compared the prevalence and risk factors of hypertension between BDHS 2011 and BDHS 2017–2018 data using multiple logistic regression model^22^. The prevalence ratio (PR) is a suitable method for cross-sectional study when the prevalence of a disease is > 10%^23,24^. Hence, the statistical modeling in this study overestimated the estimated odds ratios for the risk factors. However, we applied appropriate statistical methods to estimate the PR, which is a uniqueness of this study.

The present research was carried out to identify the change in the prevalence and associated factors of hypertension according to the ACC/AHA guidelines in comparison to that of the JNC 7 guideline using the most recent Bangladesh Demographic and Health Survey (BDHS) 2017–2018 data. This new dataset encompasses a comparatively larger measurement of BP with more participants than 2011 BDHS. Therefore, the findings using this latest dataset can broaden policy implications regarding hypertension management in Bangladesh.

## Methods

### Data sources

The study analyzed the most recent 2017–2018 BDHS dataset. The survey was carried out from October 2017 to March 2018 under the National Institute of Population Research and Training, Medical Education and Family Welfare Division, and Ministry of Health and Family Welfare. The survey’s principal objective was to assess the health indicators and provide an overview of population, maternal and child health, and the status of several NCDs such as hypertension and diabetes.

### Study population and survey design

The sampling frame used for the 2017–2018 BDHS is the complete list of enumeration areas (EAs) covering the entire population of Bangladesh. The survey used a list of enumeration areas (EAs) provided by the Bangladesh Bureau of Statistics from the 2011 Population and Housing Census of the People’s Republic of Bangladesh. The survey’s primary sampling unit (PSU) is an EA covering on average 120 households in 2017–2018. The 2017–2018 BDHS was a multistage stratified cluster sample of households’ survey, carried out in two and three stages in rural and urban settings. In the first sampling stage, rural wards were selected, following PSUs, and then households were selected from PSUs. In urban areas, wards were selected through the PSUs technique, and one EA was selected from each PSU. Then, the households were chosen from the selected EAs sample. A detailed description of the survey design, methodologies, sample size, questionnaires, and findings is available in the final summary report of 2017–2018 BDHS. Anthropometry and BP were also systematically measured from the selected subsample of 2017–2018 BDHS^25^. A total of 12,152 unweighted sample was found in the original dataset, which increased to 12,975 after applying the weight to the dataset. However, we had to exclude 1016 cases due to missing values in some of the variables, making the total weighted number of observations 11,959 for our analysis.

### Dependent variable

The dependent variable for this study was hypertension. Trained health technicians measured BP three times using LIFE SOURCE^®^ UA-767 Plus BP monitor at about ten minutes interval^25^. Then, the average of second and third measurements was used to report respondents’ final BP^25^. A person with a SBP of ≥ 140 mmHg and/or a DBP of ≥ 90 mmHg was considered hypertensive, as suggested by the JNC 7^26^. While according to the ACC/AHA 2017 guideline, individuals with a SBP of ≥ 130 mmHg and/or a DBP of ≥ 80 mmHg or who were taking any prescribed antihypertensive drugs to control BP were categorized as hypertensive^27^. The category of prehypertension was transformed into elevated blood pressure in the 2017 ACC/AHA guideline^27^.

### Explanatory variables

The explanatory variables included in the study were selected based on previous literature reporting the risk of hypertension in LMICs setting^6,11,12,28–31^. The household factors included administrative divisions (Barisal, Chittagong, Dhaka, Khulna, Rajshahi, Rangpur, Sylhet, Mymensingh); place of residence (urban, rural); and wealth status (poorest, poorer, middle, richer, richest), whereas the socioeconomic and individual factors included: age of the participants in years (18–24, 25–34, 35–44, 45–54, 55–64, ≥ 65); sex of the participants (male, female); education level (no education, primary, secondary, higher); and occupational status (not working, working). Behavioral characteristics included smoking habit (no, yes) and body mass index (BMI) level. We have used global cut-off points for BMI classification: underweight (< 18.5 kg/m^2^), normal (18.5–25.0 kg/m^2^), overweight (25.1–29.9 kg/m^2^), and obese (≥ 30.0 kg/m^2^)^32^.

### Statistical analysis

Considering the complex survey of BDHS, we prepared the data using the survey weights before the analysis. Next, the normality assumption of continuous variables was investigated from their distribution, and it was reported in the paper with medians and interquartile ranges (IQRs). Then, we estimated the prevalence of hypertension and reported the differences between the two guidelines. We reported the prevalence of hypertension by background characteristics accounting for complex survey design/survey weight. It is notable to mention that survey weights only account for the sampling scheme. Therefore, we standardized the prevalence of hypertension for the same standard population to remove or minimize the impact of differences in participant’s age and sex distribution. On the next step, we fitted the selected explanatory variables in unadjusted log-binomial regression model. Thereafter, we executed a log-binomial regression model considering survey weights including the explanatory variables having p-values (< 0.05) in the unadjusted analysis to identify the factors associated with hypertension, describing results with prevalence ratios (PRs) and their 95% confidence intervals (CIs) and p-values. We used Statistical Package for Social Science (SPSS) version 26 and SAS^®^ OnDemand for Academics for data analysis.

### Ethical consideration

The study used secondary data from the Demographic and Health Surveys (DHS) Program publicly available; therefore, we did not require any further ethical approval. The details of ethical procedures followed by the DHS Program can be found in the BDHS report^25^.

## Results

A total of 11,959 weighted participants were included in the analysis (Table 1). The median (IQR) age of the respondents was 34.0 years (18.0–95.0). Of the total participants, 6835 (57.2%) were female (Table 1). The median (IQR) of SBP and DBP were 118 mmHg (109–131) and 80 mmHg (73–87), respectively. Most of the participants (75.8%) have their BP measured at least once. The median BMI was 21.91 kg/m^2^ (19.4–24.9). In addition, 25.4% had no education, and 73.4% were residing in rural areas.

**Table 1:** Distribution of respondents by background characteristics.

Background characteristics	BDHS 2017–2018		
	All participants (N = 11,959), Frequency (%)	Hypertensive participants per JNC 7 (n = 2866), frequency (%)	Hypertensive participants per 2017 ACC/AHA (n = 6044), frequency (%)
SBP, median (IQR), ( mmHg)	118 (109–131)	109 (103–116)	114 (106–122)
DBP, median (IQR), ( mmHg)	80 (73–87)	73 (69–77)	77 (71.0–82.0)
Ever measured BP	9063 (75.8)	2379 (26.2)	4795 (52.9)
Know about hypertension status	1534 (12.8)	988 (64.4)	1286 (83.8)
Taking antihypertensive medication for BP	1216 (10.2)	812 (66.8)	1049 (86.3)
**Administrative divisions**			
Barisal	659 (5.5)	184 (27.9)	349 (53.0)
Chittagong	2057 (17.2)	507 (24.6)	1065 (51.8)
Dhaka	2770 (23.2)	564 (20.4)	1256 (45.4)
Khulna	1488 (12.4)	399 (26.8)	833 (56.0)
Rajshahi	1729 (14.5)	440 (25.4)	908 (52.5)
Rangpur	1503 (12.6)	424 (28.2)	829 (55.2)
Sylhet	780 (6.5)	165 (21.2)	366 (46.9)
Mymensingh	973 (8.1)	183 (18.8)	438 (45.0)
**Place of residence**			
Urban	3180 (26.6)	767 (24.1)	1674 (52.6)
Rural	8779 (73.4)	2099 (23.9)	4370 (49.8)
**Sex of the participants**			
Male	5124 (42.8)	1221 (23.8)	2667 (52.0)
Female	6835 (57.2)	1646 (24.1)	3376 (49.4)
**Age of the participants (years)**			
Median (IQR)	34.0 (18.0–95.0)	29.0(22.0–40.0)	31.0 (23.0–43.0)
18–24	2422 (20.3)	184 (7.6)	744 (30.7)
25–34	2972 (24.9)	412 (13.9)	1284 (43.2)
35–44	2417 (20.2)	612 (25.3)	1326 (54.8)
45–54	1672 (14.0)	564 (33.8)	1047 (62.6)
55–64	1350 (11.3)	533 (38.5)	860 (63.7)
≥ 65	1126 (9.4)	562 (48.9)	782 (69.4)
**BMI level**			
Median (IQR)	21.91 (19.4–24.9)	20.84 (18.73–23.55)	21.44 (19.13–24.36)
Underweight (< 18.5 kg/m^2^)	2071 (17.3)	338 (16.3)	723 (34.9)
Normal (18.5–25.0 kg/m^2^)	7010 (58.6)	1482 (21.1)	3342 (47.7)
Overweight (25.1–29.9 kg/m^2^)	2389 (20.0)	868 (36.3)	1620 (67.8)
Obesity (≥ 30.0 kg/m^2^)	489 (4.1)	178 (36.3)	359 (73.4)
**Education level**			
No education	3032 (25.4)	977 (32.2)	1753 (57.8)
Primary	3591 (30.0)	823 (22.9)	1754 (48.8)
Secondary	3545 (29.6)	710 (20.0)	1675 (47.2)
Higher	1790 (15.0)	355 (19.8)	862 (48.2)
**Occupational Status**			
Not working	4619 (38.6)	1213 (26.3)	2340 (50.7)
Working	7340 (61.4)	1653 (22.5)	3704 (50.5)
**Wealth status**			
Poorest	2315 (19.4)	500 (21.6)	1078 (46.6)
Poorer	2354 (19.7)	526 (22.3)	1088 (46.2)
Middle	2468 (20.6)	582 (23.6)	1266 (51.3)
Richer	2379 (19.9)	568 (23.9)	1208 (50.8)
Richest	2442 (20.4)	690 (28.3)	1404 (57.5)
**Smoking habit**			
No	10,271 (85.9)	2405 (23.4)	5140 (50.0)
Yes	1688 (14.1)	461 (27.3)	904 (53.6)

*SBP* systolic blood pressure, *DBP* diastolic blood pressure, *BP* blood pressure, *IQR* inter-quartile range, *BMI* body mass index.

Table 2 summarizes the prevalence of hypertension among the participants according to JNC 7 and ACC/AHA guideline, along with the differences between these two guidelines. Prevalence of hypertension in urban areas was 25.1% (95% CI 22.4–25.6%) in the JNC 7 and 54.0% (95% CI 52.5–55.5%) in the ACC/AHA guideline, making the difference of more than twice between them. As per JNC 7, the prevalence of hypertension among women was 24.6% (95% CI 23.1–25.6%), and it was 24.3% (95% CI 23.1–25.4%) among men, which increased considerably when the new guideline was applied. A similar result was also observed for other explanatory variables (i.e., administrative divisions, age of the participants, BMI level, education level, occupational status, wealth status, and smoking habit).

**Table 2:** Weighted prevalence of hypertension according to selected demographic characteristics.

Explanatory variables	BDHS 2017–2018		
	Prevalence of hypertension per JNC 7, prevalence (95% CI)	Prevalence of hypertension per 2017 ACC/AHA, prevalence (95% CI)	Difference, prevalence (95% CI)
**Administrative divisions**			
Barisal	27.8 (25.3–30.2)	53.5 (50.7–56.2)	25.7 (25.4–26.0)
Chittagong	24.9 (22.8–27.0)	52.6 (50.2–55.1)	27.7 (27.4–28.1)
Dhaka	20.5 (18.5–22.5)	45.5 (43.1–48.0)	25.1 (24.6–25.5)
Khulna	26.9 (24.8–29.1)	56.2 (53.8–58.6)	29.3 (25.8–29.5)
Rajshahi	25.8 (23.6–27.9)	52.8 (50.3–55.3)	26.4 (25.8–26.9)
Rangpur	28.8 (26.6–31.0)	56.3 (53.8–58.7)	27.0 (26.7–27.3)
Sylhet	21.1 (19.0–23.3)	47.6 (45.0–50.2)	27.5 (27.3–27.7)
Mymensingh	19.5 (17.5–21.6)	45.9 (43.3–48.6)	26.5 (26.0–27.0)
**Place of residence**			
Urban	25.1 (23.8–26.4)	54.0 (52.5–55.5)	28.9 (28.7–29.1)
Rural	24.1 (23.2–25.1)	50.0 (48.9–51.1)	25.9 (25.7–26.0)
**Sex of the participants**			
Male	24.3 (23.1–25.4)	52.6 (51.3–54.0)	28.4 (28.2–28.6)
Female	24.6 (23.6–25.6)	50.5 (49.3–51.7)	25.9 (25.7–26.0)
**Age of the participants (years)**			
18–24	7.8 (6.7–8.9)	31.9 (30.0–33.7)	24.1 (23.3–24.9)
25–34	14.2 (12.9–15.4)	43.7 (41.9–45.5)	29.5 (29.0–30.0)
35–44	25.5 (23.8–27.3)	55.5 (53.5–57.5)	30.0 (29.7–30.2)
45–54	34.1 (31.8–36.3)	63.7 (61.4–65.9)	29.6 (29.6–29.6)
55–64	40.9 (38.3–43.5)	64.8 (62.3–67.3)	23.9 (24.0–23.9)
≥ 65	50.3 (47.5–53.2)	70.0 (67.4–72.7)	19.7 (19.9–19.4)
**BMI level**			
Underweight (< 18.5 kg/m^2^)	16.8 (15.2–18.4)	35.4 (33.3–37.4)	18.6 (18.2–19.1)
Normal (18.5–25.0 kg/m^2^)	21.7 (20.7–22.7)	48.9 (47.7–50.1)	27.2 (27.0–27.4)
Overweight (25.1–29.9 kg/m^2^)	36.5 (34.6–38.4)	67.9 (66.0–69.7)	31.4 (31.5–31.4)
Obesity (≥ 30.0 kg/m^2^)	36.9 (32.7–41.1)	73.4 (69.5–77.2)	36.5 (36.8–36.1)
**Education level**			
No education	32.6 (30.9–34.3)	58.0 (56.2–59.7)	25.4 (25.3–25.5)
Primary	23.6 (22.2–24.9)	49.5 (47.9–51.1)	25.9 (25.7–26.2)
Secondary	20.6 (19.3–21.9)	48.4 (46.7–50.0)	27.7 (27.4–28.1)
Higher	20.9 (19.1–22.7)	50.7 (48.5–52.9)	29.9 (29.4–30.3)
**Occupational status**			
Not working	27.2 (25.9–28.4)	52.2 (50.8–53.6)	25.0 (24.9–25.2)
Working	22.7 (21.8–23.7)	50.9 (49.8–52.1)	28.2 (28.0–28.4)
**Wealth status**			
Poorest	21.0 (19.4–22.6)	45.6 (43.6–47.6)	24.6 (24.2–25.0)
Poorer	22.3 (20.6–24.0)	46.7 (44.7–48.7)	24.4 (24.1–24.7)
Middle	24.1 (22.4–25.8)	51.9 (49.9–53.9)	27.7 (27.5–28.0)
Richer	25.3 (23.5–27.0)	52.8 (50.8–54.8)	27.5 (27.3–27.8)
Richest	28.9 (27.2–30.7)	59.0 (57.1–60.8)	30.0 (29.9–30.2)
**Smoking habit**			
No	23.9 (23.0–24.7)	51.0 (50.0–51.9)	27.1 (27.0–27.3)
Yes	27.9 (25.8–29.9)	53.9 (51.6–56.2)	26.1 (25.8–26.3)

*CI* confidence interval, *BMI* body mass index.

Table 3 describes the risk factors associated with hypertension under JNC 7 and ACC/AHA guidelines after adjusting the explanatory variables in the log-binomial multivariate model setting. Advanced age, increased BMI, and participants from Rangpur and Rajshahi divisions had higher PRs as per both guidelines, indicating an increased risk of hypertension. Alternatively, smoking had a significantly lower impact on hypertension only in JNC 7, but the result is insignificant for ACC/AHA guideline. To note, before fitting explanatory variables in the log-binomial multivariate setting, we checked unadjusted log-binomial models and found that participants’ age, place of residence, and occupational status had an insignificant impact on hypertension in one of the guidelines (Supplementary Table 1). Thus, we did not include these variables while multivariate modeling.

**Table 3:** Factors associated with hypertension in according to selected demographic characteristics.

Explanatory variables	BDHS 2017–2018			
	JNC 7		ACC/AHA 2017	
	PR (95% CI)	p-value	PR (95% CI)	p-value
**Administrative divisions**				
Dhaka (RC)	1		1	
Barisal	1.30 (1.15–1.48)	< 0.0001	1.08 (1.03–1.13)	0.0032
Chittagong	1.14 (1.04–1.26)	0.0054	1.05 (1.02–1.09)	0.0037
Khulna	1.17 (1.06–1.30)	0.0017	1.08 (1.04–1.12)	< 0.0001
Mymensingh	0.94 (0.81–1.08)	0.3824	1.01 (0.97–1.07)	0.4432
Rajshahi	1.22 (1.10–1.34)	< 0.0001	1.08 (1.03–1.12)	< 0.0001
Rangpur	1.32 (1.20–1.46)	< 0.0001	1.11 (1.07–1.15)	< 0.0001
Sylhet	1.12 (0.98–1.29)	0.0977	1.05 (1.00–1.11)	0.0585
**Age of the participants (years)**				
18–24 (RC)	1		1	
25–34	1.66 (1.41–1.96)	< 0.0001	1.10 (1.06–1.15)	< 0.0001
35–44	2.99 (2.56–3.50)	< 0.0001	1.23 (1.18–1.28)	< 0.0001
45–54	4.10 (3.51–4.81)	< 0.0001	1.33 (1.27–1.38)	< 0.0001
55–64	5.01 (4.28–5.87)	< 0.0001	1.37 (1.31–1.43)	< 0.0001
≥ 65	6.30 (5.40–7.36)	< 0.0001	1.44 (1.38–1.51)	< 0.0001
**BMI level**				
Normal (18.5–25.0 kg/m^2^) (RC)	1		1	
Underweight (< 18.5 kg/m^2^)	0.71 (0.64–0.78)	< 0.0001	0.86 (0.83–0.89)	< 0.0001
Overweight (25.1–29.9 kg/m^2^)	1.59 (1.48–1.69)	< 0.0001	1.17 (1.15–1.20)	< 0.0001
Obesity (≥ 30.0 kg/m^2^)	1.86 (1.41–1.96)	< 0.0001	1.21 (1.17–1.26)	< 0.0001
**Education level**				
Secondary education (RC)	1		1	
No education	1.01 (0.93–1.10)	0.7623	1.01 (0.98–1.05)	0.3774
Primary	0.95 (0.88–1.02)	0.1988	0.94 (0.86–1.02)	0.4672
Higher	0.96 (0.88–1.06)	0.5193	0.96 (0.97–1.04)	0.7753
**Wealth status**				
Middle (RC)	1		1	
Poorer	0.98 (0.89–1.07)	0.6251	0.96 (0.93–0.99)	0.0275
Poorest	0.99 (0.90–1.09)	0.7992	0.98 (0.94–1.01)	0.1698
Richer	0.99 (0.91–1.08)	0.8888	1.00 (0.96–1.03)	0.8332
Richest	1.05 (0.96–1.15)	0.2968	1.02 (0.98–1.05)	0.3906
**Smoking habit**				
No (RC)	1		1	
Yes	0.88 (0.81–0.95)	0.0014	0.97 (0.95–1.01)	0.1161

*PR* prevalence ratio, *CI* confidence interval, *p-value* probability value, *RC* reference category, *BMI* body mass index.

**Figure 1:**
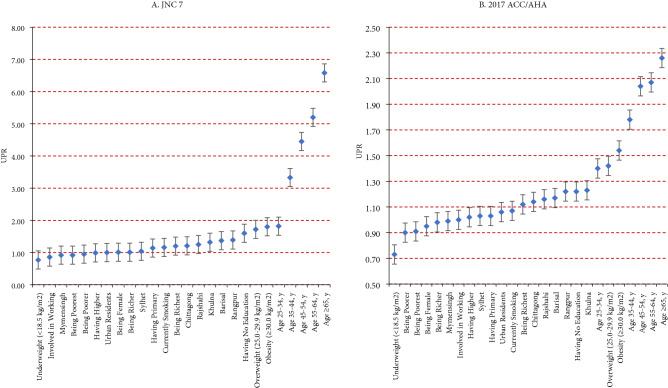
Unadjusted prevalence ratio (UPR) of factors associated with hypertension according to JNC 7 and 2017 ACC/AHA.

Figure 1 exerts the results of single-adjusted models where people age (≥ 65) showed the strongest association with developing hypertension (UPR = 6.58, 95% CI 5.66–7.65, p < 0.001) followed by age (55–64; 45–54 and 35–44) showed greater odds of having hypertensive according to JNC 7 guidelines. Whereas the magnitude of associations as per 2017 ACC/AHA guidelines shows the highest odds age (≥ 65), followed by age bracket (55–64 and 45–54), obese people have had the highest odds of having hypertensive (UPR = 2.26, 95% CI 2.10–2.43, p < 0.001).

## Discussion

The current study presented unique findings based on recently published BDHS data released by the government of Bangladesh in 2020 under DHS program. A few studies examined the prevalence of hypertension according to the new guideline and have compared the results with previous JNC 7 guidelines^6,12^. These studies found an absolute change in hypertension prevalence after applying the new 2017 ACC/AHA guidance. The benefit of early detection of hypertensive individuals would reduce hypertension-related complications and cardiovascular morbidity.

Our findings depicted the change in the estimated prevalence of hypertension in Bangladesh as per JNC 7 and 2017 ACC/AHA guidelines, developed to classify prehypertension and hypertension status in humans. Under these two guidelines, we found differences in the prevalence rate at the national and individual levels. In the year 2011, according to the new lower blood pressure threshold recommended by 2017 ACC/AHA guidelines, (43.3%) prevalence of hypertension observed in Bangladesh was higher at the national level, while (20.9%) lower prevalence was documented in the previous recommendation of JNC 7^6,33,34^. Our study showed, the prevalence of hypertension augmented alarmingly in both conditions; 24.0% according to JNC 7 and 50.5% in 2017 ACC/AHA guidelines. The prevalence of hypertension according to JNC 7 was 20.9% in 2011^6,33,34^, and at least 3.1% increase was found in our present study. Whereas, 24.0% prevalence was observed in the new 2017 ACC/AHA guideline and at least 7.2% increased prevalence of hypertension was found. In addition, regardless of the respondents’ background status, these findings show the prevalence has been increasing among female participants alarmingly based on the previous studies^6,33^. When hypertension has been classified based on the 2017 ACC/AHA guideline’s thresholds, a substantial rise of the prevalence observed for all countries such as Nepal, the USA^12,35^ and Bangladesh^11^ despite different socioeconomic characteristics. This depicts the alarming increase in the prevalence of hypertension, which requires the need of urgent attention from all the stakeholders who are interested in prevention and control of hypertension in Bangladesh.

Interestingly, our findings reported a similar prevalence of hypertension among male and female participants. If we compare the finding based on the previous study, the prevalence rate increased among female participants more than male counterparts^11,28^. The plausible explanation could be biological and behavioural characteristics among the females might have increased over the period. This fact is supported by the previous evidence that females have a higher risk of obesity and diabetes compared with men^36,37^. This needs females to require more awareness and public health information to control hypertension and minimize adverse complications^28^. Our study findings reported that people with higher socioeconomic status had higher odds of having hypertension. The higher wealth status participants can generally purchase more consumable resources with a large amount of calorie intake, making them overweight or obese, putting them at a greater risk of being hypertensive than those lower wealth status^38,39^. This suggests the need for prevention and control program for hypertension in urban areas of Bangladesh.

The prevalence of hypertension was higher among those living in urban areas, which is in line with previous studies where urban people were reported more hypertensive^6,28,40,41^. The possible reason could be prevailing unhealthy lifestyle factors such as less physical activity, consumption of unhealthy diets among the urban populations might have contributed to the disease burden^42–44^. However, this finding warrants further detailed investigation of causes for the increased prevalence or odds of hypertension in several Bangladesh divisions^28^. This finding suggests the need to understand the social inequalities among the rural and urban community, which may have played a role in such variation. Understanding the inequalities mentioned earlier may help design the comprehensive hypertension prevention and control program for Bangladesh peoples.

There is another explanation that would help understand why the prevalence of hypertension is high in urban areas. The study found that higher educated and higher wealth status of people are likely lives in urban areas, resulting from having a sedentary lifestyle such as low physical activity. A lack of open spaces for playing games or physical activity might result in the high-risk prevalence of hypertension^28^. Since most urban participants are educated, and these had a higher prevalence of hypertension. Thus our study recommends that educated individuals in urban areas need to receive more public health awareness information to control raised blood pressure levels^28^.

This study identified the potential risk factors of hypertension using both JNC 7 and 2017 ACC/AHA guideline alongside to the estimation of the prevalence. People of older age 25 to more, overweight, and obese had relatively higher odds, which is in line with previous studies elsewhere^6,12,45–47^. Notably, in the current study, administrative divisions were also found significantly associated with hypertension in line with suggest 2017 ACC/AHA guidelines. People from Rangpur and Rajshahi division found higher odds in the two guidelines. Much is unknown why the people from these two divisions owned higher risk of being hypertensive; however, the reason may be because of socioeconomic inequalities such as limited resources, income inequality, low level of education and social safety net programs, poor connectivity with the urban centres, insufficiency or absence of public infrastructure^48,49^.

The new 2017 ACC/AHA guideline recommends treating stage 1 hypertension with changing lifestyle measures and taking antihypertensive medicines to prevent future cardiovascular disease risks^12^. Our study findings are significant because it shows that above fifty per cent of adults with hypertension or elevated blood pressure according to the new 2017 ACC/AHA classification require active lifestyles and healthy dietary habits. Public health programs should adequately address this emerging problem; in Bangladesh, emphasis should be paid to prevention and self-management of a condition not only for those with hypertension but also for all adults^12^. Therefore, it is essential to estimate the prevalence based on both thresholds to control this hypertension burden, which might exacerbate cardiovascular disease. These findings might help future researchers and appropriate authority design any programs and policies regarding control and prevent hypertension burden and overcome this massive public health challenge.

The strengths and weaknesses of this study are accredited. The strength that lies in this study is the generalizability of the findings for Bangladesh since this survey covered nationally representative data covering all divisions. Along with appropriate statistical methods to estimate the weighted prevalence of hypertension from the sample.

The limitations of the study are appropriately acknowledged. Due to a cross-sectional setup, no causality cannot be established, and the individuals’ blood pressure was measured three times in a single day. However, both guidelines recommend longitudinal measurement of blood pressure levels to diagnose hypertension^12^. This survey also used an automated device, though both guidelines recommend recording blood pressure with a sphygmomanometer^33,50,51^.

## Conclusions

The present study highlighted that the prevalence of hypertension was almost doubled according to the 2017 ACC/AHA guideline compared to the JNC 7 guideline. The policymakers and public health practitioners should consider the new guideline and make new strategies to increase awareness among the adult population in Bangladesh. The study finding also points towards addressing the already established modifiable risk factors of hypertension such as overweight/obesity, high-income status, which are also identified as the risk factors according to both guidelines.

## Supplementary Information

Supplementary Table 1.
