# Supplementary material for: Prevalence of and factors associated with hypertension according to JNC 7 and ACC/AHA 2017 guidelines in Bangladesh
Source: Sci Rep. 2021 Jul 29;11:15420. doi: 10.1038/s41598-021-94947-2 (PMC8322062; doi:10.1038/s41598-021-94947-2)
Supplement: Supplementary file 1 — Supplementary Table 1. [file 41598_2021_94947_MOESM1_ESM.docx]

Prevalence of and Factors Associated with Hypertension According to JNC 7 and ACC/AHA 2017 Guidelines in Bangladesh

**First Author:**

Md. Ashfikur Rahman*

Development Studies Discipline, Social Science School, Khulna University, Khulna-9208, Bangladesh

Email: [ashfikur@](mailto:ashfikur@)ku.ac.bd

**ORCID:** <https://orcid.org/0000-0002-5517-8557>

**Second Author:**

Henry Ratul Halder

1. Statistics Discipline, Science, Engineering, and Technology (SET) School, Khulna University, Khulna-9208, Bangladesh

2. Rady Faculty of Health Sciences, Department of Community Health Sciences, University of Manitoba, Winnipeg, Manitoba, Canada

**Email:** [halderhr@myumanitoba.com](mailto:halderhr@myumanitoba.com)

**ORCID:** <https://orcid.org/0000-0002-1362-3077>

**Third Author:**

Dr. Uday Narayan Yadav

1. Forum for Health Research and Development, Dharan, Nepal

2. Centre for Primary Health Care and Equity, University of New South Wales, Sydney, Australia

**Email:** [u.yadav@unsw.edu.au](mailto:u.yadav@unsw.edu.au)

**ORCID**: <https://orcid.org/0000-0002-6626-1604>

**Fourth Author:**

Dr. Sabuj Kanti Mistry

1. BRAC James P Grant School of Public Health, BRAC University, 68 Shahid Tajuddin Ahmed Sharani, Mohakhali, Dhaka-1212, Bangladesh

2. Centre for Primary Health Care and Equity, University of New South Wales, Sydney, Australia

**Email:** [smitra411@gmail.com](mailto:sabujkanti.mistry@unsw.edu.au)

**ORCID:** <https://orcid.org/0000-0001-6100-6076>

**Supplementary Table 1,**

| **Supplementary Table 1.** Single Adjusted Model | | | | | |
| --- | --- | --- | --- | --- | --- |
| **Explanatory Variables** | | **JNC 7** | | **2017 ACC/AHA** | |
|  |  | **UPR (95% CI)** | **p-value** | **UPR (95% CI)** | **p-value** |
| **Administrative Divisions** | |  |  |  |  |
|  | Dhaka (RC) | 1 |  | 1 |  |
|  | Barisal | 1.37 (1.19-1.58) | <0.0001 | 1.17 (1.07-1.27) | 0.0002 |
|  | Chittagong | 1.21 (1.08-1.35) | 0.0004 | 1.14 (1.08-1.21) | <0.0001 |
|  | Khulna | 1.32 (1.18-1.47) | <0.0001 | 1.23 (1.16-1.31) | <0.0001 |
|  | Mymensingh | 0.92 (0.80-1.07) | 0.3027 | 0.99 (0.91-1.07) | 0.8285 |
|  | Rajshahi | 1.25 (1.12-1.39) | <0.0001 | 1.16 (1.09-1.23) | <0.0001 |
|  | Rangpur | 1.39 (1.24-1.55) | <0.0001 | 1.22 (1.14-1.29) | <0.0001 |
|  | Sylhet | 1.04 (0.89-1.21) | 0.6124 | 1.03 (0.95-1.13) | 0.4491 |
| **Place of Residence** | |  |  |  |  |
|  | Rural (RC) | 1 |  | 1 |  |
|  | Urban | 1.00 (0.94-1.08) | 0.7965 | 1.06 (1.02-1.10) | 0.0049 |
| **Sex of the Participants** | |  |  |  |  |
|  | Male (RC) | 1 |  | 1 |  |
|  | Female | 1.01 (0.95-1.08) | 0.7455 | 0.95 (0.92-0.98) | 0.0039 |
| **Age of the Participants (years)** | |  |  |  |  |
|  | 18-24 (RC) | 1 |  | 1 |  |
|  | 25-34 | 1.82 (1.55-2.16) | <0.0001 | 1.40 (1.31-1.51) | <0.0001 |
|  | 35-44 | 3.33 (2.86-3.90) | <0.0001 | 1.78 (1.66-1.91) | <0.0001 |
|  | 45-54 | 4.45 (3.82-5.20) | <0.0001 | 2.04 (1.90-2.19) | <0.0001 |
|  | 55-64 | 5.20 (4.46-6.07) | <0.0001 | 2.07 (1.93-2.23) | <0.0001 |
|  | ≥65 | 6.58 (5.66-7.65) | <0.0001 | 2.26 (2.10-2.43) | <0.0001 |
| **BMI Level** | |  |  |  |  |
|  | Normal (18.5-24.9 kg/m^2^) (RC) | 1 |  | 1 |  |
|  | Underweight (<18.5 kg/m^2^) | 0.77 (0.69-0.86) | <0.0001 | 0.73 (0.69-0.78) | <0.0001 |
|  | Overweight (25.0-29.9 kg/m^2^) | 1.72 (1.60-1.84) | <0.0001 | 1.42 (1.37-1.48) | <0.0001 |
|  | Obesity (≥30.0 kg/m^2^) | 1.80 (1.51-1.95) | <0.0001 | 1.54 (1.45-1.63) | <0.0001 |
| **Education Level** | |  |  |  |  |
|  | Secondary Education (RC) | 1 |  | 1 |  |
|  | No Education | 1.60 (1.48-1.75) | <0.0001 | 1.22 (1.17-1.28) | <0.0001 |
|  | Primary | 1.14 (1.05-1.25) | 0.0030 | 1.03 (0.99-1.08) | 0.1763 |
|  | Higher | 0.99 (0.88-1.11) | 0.8772 | 1.02 (0.96-1.08) | 0.5307 |
| **Occupational Status** | |  |  |  |  |
|  | Not Working (RC) | 1 |  | 1 |  |
|  | Working | 0.86 (0.80-0.91) | <0.0001 | 1.00 (0.96-1.03) | 0.8420 |
| **Wealth Status** | |  |  |  |  |
|  | Middle (RC) | 1 |  | 1 |  |
|  | Poorer | 0.95 (0.85-1.05) | 0.3144 | 0.90 (0.85-0.96) | 0.0005 |
|  | Poorest | 0.92 (0.83-1.02) | 0.1051 | 0.91 (0.86-0.96) | 0.0011 |
|  | Richer | 1.01 (0.92-1.12) | 0.8068 | 0.98 (0.94-1.05) | 0.7174 |
|  | Richest | 1.20 (1.09-1.32) | 0.0002 | 1.12 (1.07-1.18) | <0.0001 |
| **Smoking Habit** | |  |  |  |  |
|  | No (RC) | 1 |  | 1 |  |
|  | Yes | 1.16 (1.07-1.27) | 0.0004 | 1.07 (1.02-1.12) | 0.0064 |
| UPR: Unadjusted prevalence ratio; CI: Confidence interval; p-value: probability value; RC: Reference category; BMI: Body mass index. | | | | | |
